# Courageous leadership and nurses’ speaking-up' behavior - the indirect roles of just culture and psychological safety: a cross-sectional study

**DOI:** 10.1016/j.ijnsa.2026.100663

**Published:** 2026-08-16

**Authors:** Ali D. Abousoliman, Heba Gamal Elgamal

**Affiliations:** a Assistant Professor of Nursing Administration & Management, College of Nursing, Prince Sattam bin Abdulaziz University, Al-Kharj city, Saudi Arabia; b Lecturer of Nursing Administration, Faculty of Nursing, KafrElsheikh University, Egypt

**Keywords:** Courageous leadership, Just culture, Psychological safety, Speaking-up behavior, Nurses, Patient safety

## Abstract

**Background:**

**:** Nurses’ speaking-up behavior is essential for preventing patient harm, yet it is shaped by leadership practices, organizational culture, and team climate. Courageous leadership may encourage nurses to voice patient safety concerns by supporting fair responses to errors and psychologically safe work environments.

**Aim:**

**:** This study examined the association between courageous leadership and nurses’ speaking-up behavior and explored the indirect roles of just culture and psychological safety among clinical nurses in Port Said, Egypt.

**Methods:**

**:** A quantitative, descriptive, cross-sectional correlational design was used. Data were collected from 288 clinical nurses working in thirteen hospitals in Port Said, Egypt, using structured self-administered questionnaires. The study variables were courageous leadership, just culture, psychological safety, and speaking-up behavior. Data were analyzed using descriptive statistics, independent-samples t-tests, one-way analysis of variance, Pearson’s correlation, and PROCESS Macro Model 6 with 5,000 bootstrap samples and HC3 heteroskedasticity-consistent standard errors.

**Results:**

**:** Courageous leadership was positively correlated with just culture, psychological safety, and speaking-up behavior. Just culture was strongly correlated with psychological safety and weakly correlated with speaking-up behavior, while psychological safety was not significantly correlated with speaking-up behavior. In the serial indirect pathway analysis, courageous leadership remained significantly and directly associated with speaking-up behavior. However, neither just culture nor psychological safety showed a significant indirect role in this association. The serial indirect pathway through just culture and psychological safety was also not significant. All bootstrap confidence intervals for the indirect effects included zero.

**Conclusion:**

**:** The findings therefore indicate that courageous leadership was directly associated with speaking-up behavior, while the observed association was not statistically accounted for by the proposed indirect pathways through just culture and psychological safety.

**What is known**

• Nurses’ speaking-up behavior is essential for identifying unsafe practices and preventing patient harm.
• Leadership behavior, just culture, and psychological safety may influence whether nurses feel able to raise patient-safety concerns.
• Evidence examining courageous leadership, just culture, psychological safety, and speaking-up behavior within one theoretically ordered model remains limited, particularly in Egyptian healthcare settings.

**What this paper adds**

• Nurses who perceived greater courageous leadership in their immediate nurse managers reported more frequent speaking-up behavior.
• Courageous leadership was positively associated with perceived just culture and psychological safety, while just culture was strongly associated with psychological safety.
• Neither just culture nor psychological safety statistically accounted for the association between courageous leadership and speaking-up behavior, highlighting the importance of direct leadership behaviors that invite, reinforce, and protect nurses’ voices.

## 1 Introduction

Patient safety remains a major global concern, with preventable adverse events continuing to harm hospitalized patients and contribute to avoidable morbidity and mortality worldwide (World Health Organization [WHO], 2021). Nurses’ speaking-up behavior, defined here as verbally communicating concerns about errors, unsafe practices, or patient-safety risks to a person who can take corrective action, is an essential safeguard against preventable harm because nurses’ continuous proximity to patients places them in a strong position to identify emerging risks and unsafe clinical conditions (Hoffmann et al., 2022; Jeong & Kim, 2023; Lee & Kwon, 2024). However, nurses may remain silent when they anticipate blame, punishment, hierarchical pressure, poor managerial response, or negative professional consequences (Badran et al., 2026; Malik et al., 2024; Yilmaz, 2025).

In hospital settings, speaking up is not only an individual behavior but also a discretionary safety behavior shaped by nurses’ judgments about whether voicing a concern will be effective and whether doing so will expose them to interpersonal or professional risk. Leadership behavior, organizational responses to errors, and the immediate team climate may therefore either facilitate or inhibit speaking up. In many healthcare contexts, particularly in settings where hierarchical communication and blame-oriented responses to errors may exist, nurses may hesitate to report concerns or challenge unsafe practices. Therefore, understanding the organizational and team-level conditions that encourage nurses to speak up is important for improving patient safety. In Egypt, evidence examining how perceived leadership behavior, just culture, and psychological safety are jointly associated with nurses’ speaking-up behavior remains limited, particularly in studies involving hospitals from different healthcare sectors and examining these factors within a single model. This gap highlights the need to examine factors that may be associated with nurses’ willingness to communicate patient safety concerns in Egyptian clinical settings.

Leadership is central to shaping whether clinical environments encourage or suppress open communication. In this study, perceived courageous leadership refers to nurses’ perceptions that their immediate nurse manager acts according to ethical principles, addresses difficult or unsafe situations, and accepts possible personal or professional risk when action is required (Bangari & Prasad, 2012; Huang et al., 2025). Although courageous leadership may overlap with ethical, authentic, or transformational leadership, its distinguishing feature is the leader’s willingness to confront unsafe or unethical practices despite the potential personal or professional consequences of doing so**.** In nursing, perceived courageous leadership may support safety communication by demonstrating moral action, challenging unsafe practices, responding constructively to concerns, and signaling that nurses’ voices will be taken seriously (Althobaiti, 2026; Meng & Guo, 2025; Mohammadi et al., 2022; Wang et al., 2024; Yılmaz & Özbek Güven, 2024). Recent evidence also suggests that supportive and ethically grounded leadership behaviors are associated with nurses’ moral courage and psychological safety, both of which are relevant to patient safety communication (Lee & Cho, 2026).

Two important organizational and team-level conditions may help explain the association between perceived courageous leadership and nurses’ speaking-up behavior: just culture and psychological safety. Just culture reflects a fair and learning-oriented approach to error management that balances accountability with system improvement rather than blame (Marx, 2001; Petschonek et al., 2013; Robichaux & Vittone, 2023). Psychological safety refers to the shared belief that team members can ask questions, admit mistakes, and voice concerns without fear of embarrassment, rejection, or punishment (Edmondson, 1999; Ito et al., 2022). Although these concepts are related, they represent different levels of the safety environment. Perceived just culture concerns nurses’ views of the broader organizational approach to fairness, accountability, and learning from errors, whereas psychological safety concerns the immediate team climate for interpersonal risk-taking. A hospital may formally promote fair and learning-oriented responses to errors, but nurses may not necessarily feel psychologically safe within their immediate clinical teams. Distinguishing the two constructs is therefore important when examining how organizational conditions may be associated with speaking-up behavior.

A social-information-processing perspective provides a theoretical rationale for the proposed sequence because employees use leaders’ actions and organizational responses as cues when interpreting which behaviors are expected, supported, or risky within the workplace (Salancik & Pfeffer, 1978). Perceived courageous leadership may be associated with stronger perceptions of just culture because courageous leaders are expected to respond fairly to errors, challenge blame-oriented practices, and promote learning from unsafe events. In turn, a stronger just culture may be associated with higher psychological safety because nurses who perceive fairness and learning-oriented responses to error may feel safer expressing concerns within their teams. Psychological safety may then be associated with greater speaking-up behavior because nurses are more likely to voice patient safety concerns when they believe they will not be blamed, embarrassed, or punished. Evidence shows that just culture and psychological safety are associated with reduced silence, safety communication, voice behavior, and safety improvement in healthcare settings (Bahadurzada et al., 2024; Ibrahim El-Sayed et al., 2024; O’Donovan et al., 2021; Silva et al., 2025; Weiss et al., 2023). However, because these constructs were measured at one time point in the present study, the proposed sequence represents a theoretically ordered statistical model rather than an established temporal or causal process.

Although courageous leadership, just culture, psychological safety, and speaking-up behavior have been examined separately, few studies have examined them together within a theoretically ordered model, and evidence involving Egyptian clinical nurses remains particularly limited. Examining these variables jointly may clarify whether nurses’ perceptions of courageous leadership are associated with speaking-up behavior directly and through distinct organizational and team-level conditions.

### 1.1 Aim of the study

This study aimed to examine the association between nurses’ perceptions of their immediate nurse manager’s courageous leadership and nurses’ speaking-up behavior and to estimate the statistical indirect associations through perceived just culture and psychological safety, both separately and sequentially, among clinical nurses in hospitals in Port Said, Egypt.

### 1.2 Study objectives

The specific objectives were to:

• Describe nurses’ perceptions of their immediate nurse manager’s courageous leadership, just culture, psychological safety, and self-reported speaking-up behavior.
• Estimate the bivariate associations among perceived courageous leadership, just culture, psychological safety, and speaking-up behavior.
• Estimate the direct statistical association between perceived courageous leadership and nurses’ speaking-up behavior.
• Estimate the specific indirect associations between perceived courageous leadership and speaking-up behavior through just culture and psychological safety separately.
• Estimate the prespecified serial indirect association between perceived courageous leadership and speaking-up behavior through just culture followed by psychological safety.

### 1.3 Literature review and hypothesis development

Speaking-up behavior is an important component of patient safety because it enables nurses to report errors, question unsafe decisions, and communicate risks before patient harm occurs (Hoffmann et al., 2022; WHO, 2021). In the present study, speaking up refers specifically to directly communicating a patient-safety concern to a person who may be able to take corrective action. It is conceptually distinct from formal incident reporting, although both represent important forms of patient-safety communication. Nurses are often in continuous contact with patients and may be the first healthcare professionals to detect early signs of unsafe care. However, speaking up is a discretionary behavior that depends partly on nurses’ judgments about whether voicing a concern will be effective and whether doing so will expose them to interpersonal or professional risk. Nurses may hesitate to voice concerns when they anticipate blame, interpersonal conflict, managerial inaction, punishment, or negative professional consequences (Malik et al., 2024; Yilmaz, 2025). Previous studies have shown that speaking-up behavior is associated with organizational culture, teamwork, working conditions, and previous experiences of communicating patient-safety concerns (Jeong & Kim, 2023; Lee & Kwon, 2024). Training interventions have also been used to strengthen speaking-up behavior among nurses (Hur et al., 2025). However, skills-based interventions alone may be insufficient when leadership responses, organizational practices, or team interactions signal that raising concerns is unsafe or unlikely to produce corrective action.

Perceived courageous leadership may be particularly relevant to nurses’ speaking-up behavior because it reflects nurses’ judgments that their immediate nurse manager is willing to act ethically, confront difficult situations, and address unsafe practices despite possible personal or professional consequences. Courageous leaders are expected to challenge harmful practices, protect staff and patients, and support morally responsible action (Bangari & Prasad, 2012; Huang et al., 2025). Courageous leadership overlaps with ethical, authentic, and transformational leadership in its emphasis on values and responsible action; however, its proposed distinguishing feature is the leader’s willingness to confront unsafe or unethical practices despite the risks associated with doing so. In nursing and healthcare settings, related evidence indicates that ethical leadership, moral courage, and moral sensitivity are associated with safer care, whistleblowing intentions or behaviors, and stronger safety-related behaviors among nurses (Meng & Guo, 2025; Mohammadi et al., 2022; Wang et al., 2024; Yılmaz & Özbek Güven, 2024). Leadership has also been identified as an important correlate of patient-safety culture in healthcare organizations (Althobaiti, 2026). Although these related leadership and moral constructs are not equivalent to courageous leadership, they provide a theoretical basis for expecting nurses who perceive their managers as courageous to be more willing to communicate patient-safety concerns. Direct evidence examining courageous leadership and nurses’ speaking-up behavior nevertheless remains limited.

Just culture provides an organizational foundation for speaking up by promoting fairness, balanced accountability, trust, open communication, and learning from errors rather than blame (Marx, 2001; Petschonek et al., 2013; Robichaux & Vittone, 2023). A just culture does not imply the absence of individual accountability; rather, it differentiates among human error, risky behavior, and reckless conduct while emphasizing organizational learning and system improvement. When nurses perceive that errors and safety concerns are handled fairly, they may become more willing to report incidents and discuss unsafe conditions. Recent studies have linked just culture with reduced patient-safety silence, improved error reporting, and more constructive responses to safety incidents among nurses (Badran et al., 2026; Ibrahim El-Sayed et al., 2024; Jeong et al., 2025). Although just culture is conceptually an organizational characteristic, the present study examines nurses’ individual perceptions of that culture. Accordingly, the construct is referred to as perceived just culture when interpreting the study findings. Perceived courageous leadership may be associated with stronger perceptions of just culture because courageous leaders may challenge blame-oriented responses, encourage fair accountability, and support learning from adverse events. Their responses to reported errors and safety concerns may serve as visible signals of whether organizational commitments to fairness and learning are enacted in daily clinical practice.

Psychological safety represents the team-level condition that allows nurses to take interpersonal risks, such as asking questions, admitting mistakes, challenging unsafe decisions, and speaking up about concerns (Edmondson, 1999; Ito et al., 2022). Studies in healthcare settings have shown that psychological safety is positively associated with voice behavior, joint problem-solving, safety improvement, and perceived team effectiveness (Bahadurzada et al., 2024; O’Donovan et al., 2021; Weiss et al., 2023). Quality-improvement work also suggests that efforts to strengthen psychological safety can support speaking up and safety-event reporting (Silva et al., 2025). In addition, psychological safety may help clarify how leadership is associated with nurses’ moral courage and safety-related communication (Lee & Cho, 2026). Managers’ responses to questions, mistakes, and expressions of disagreement provide interpersonal cues about whether speaking up will be welcomed or sanctioned. Nurses who perceive their managers as willing to address difficult safety issues may therefore report greater psychological safety and a greater willingness to raise concerns. Although psychological safety is theoretically conceptualized as a shared team belief, the present study measures individual nurses’ perceptions of psychological safety; team-level interpretations would require evidence that individual responses can appropriately be aggregated.

Although just culture and psychological safety are related, they represent distinct aspects of the safety environment. Just culture reflects the broader organizational approach to fairness, accountability, and error management, whereas psychological safety reflects the immediate team climate that enables interpersonal risk-taking. Perceived just culture may be directly associated with speaking-up behavior because expectations of fair treatment can reduce nurses’ anticipated costs of raising a concern. It may also be indirectly associated with speaking up through psychological safety. Nurses who perceive a fair and learning-oriented organizational culture may feel safer within their teams to express concerns, ask questions, and acknowledge mistakes. Thus, just culture and psychological safety should not be treated as interchangeable constructs: an organization may have formal policies supporting fair error management while individual nurses still feel unable to challenge colleagues or managers within their immediate teams.

Social information processing theory provides a framework for the proposed relationships. The theory suggests that employees interpret social and organizational cues when deciding which behaviors are expected, supported, or potentially risky in the workplace (Salancik & Pfeffer, 1978). Within clinical environments, nurses may use their managers’ actions and organizational responses to errors as information about the likely consequences of speaking up. Perceived courageous leadership may provide an initial leadership-level signal that patient-safety concerns will be acknowledged and addressed. Fair and learning-oriented responses to errors may subsequently strengthen perceptions of just culture, while those perceptions may contribute to an immediate team environment in which interpersonal risk-taking feels safer. Psychological safety may then be more proximally associated with the decision to voice a patient-safety concern.

Although courageous leadership, just culture, psychological safety, and speaking-up behavior have been examined separately, previous studies have more commonly investigated isolated or pairwise relationships than all four constructs within one theoretically ordered model. Evidence examining these relationships among Egyptian clinical nurses and across different hospital sectors also remains limited. Based on the available evidence and social information processing theory, the present study tested whether perceived courageous leadership was associated with nurses’ speaking-up behavior directly and through perceived just culture and psychological safety. The proposed order places perceived just culture before psychological safety because organizational expectations of fairness and learning may shape whether nurses experience their immediate teams as safe environments for interpersonal risk-taking. However, because all variables were measured at one time point, the serial order represents a theoretically specified statistical model and should not be interpreted as demonstrating a temporal or causal mechanism. Reverse and alternative associations cannot be excluded.

### 1.4 Hypotheses

H1a: Perceived courageous leadership is positively associated with nurses’ speaking-up behavior.

H1b: Perceived courageous leadership is positively associated with perceived just culture.

H1c: Perceived courageous leadership is positively associated with perceived psychological safety.

H2: Perceived just culture is positively associated with perceived psychological safety.

H3a: Perceived just culture is positively associated with nurses’ speaking-up behavior.

H3b: Perceived psychological safety is positively associated with nurses’ speaking-up behavior.

H4: The specific indirect association between perceived courageous leadership and nurses’ speaking-up behavior through perceived just culture is positive.

H5: The specific indirect association between perceived courageous leadership and nurses’ speaking-up behavior through perceived psychological safety is positive.

H6: The specific serial indirect association between perceived courageous leadership and nurses’ speaking-up behavior through perceived just culture followed by perceived psychological safety is positive.

## 2 Methods

### 2.1 Study design

This study used a quantitative, cross-sectional correlational survey design. The design was used to estimate concurrent associations among perceived courageous leadership, perceived just culture, psychological safety, and nurses’ speaking-up behavior and to examine prespecified statistical indirect associations among these variables. Because the exposure, proposed intervening variables, and outcome were measured at the same time, temporal precedence and causal mediation could not be established. Accordingly, the findings were interpreted as cross-sectional associations rather than causal effects or confirmed mechanisms.

### 2.2 Setting

The study was conducted in 13 hospitals in Port Said, Egypt, between February 3, 2026, and February 26, 2026. These hospitals included six hospitals affiliated with the Ministry of Health, two hospitals affiliated with the Health Insurance Organization, and five private hospitals. The hospitals therefore represented three healthcare sectors operating within Port Said.

The selected hospitals provided a range of adult inpatient services, including medical, surgical, intensive care, gynecology, obstetrics, orthopedic, burns, and open-heart care units. The hospitals were purposively selected because they provided multiple adult inpatient services and granted permission for data collection. Their inclusion was not intended to produce a probability sample of all hospitals in Port Said or Egypt. Adult inpatient settings were included because nurses working in these units are regularly exposed to clinical situations requiring patient-safety communication, error reporting, and speaking-up about unsafe practices.

### 2.3 Participants

The study population consisted of clinical nurses providing direct patient care in the selected hospitals. Nurses were eligible to participate if they held a valid Egyptian nursing license, voluntarily consented to participate, had worked in their current hospital for at least six months, and were assigned to an adult inpatient unit during the data-collection period. The six-month criterion was intended to provide participants with sufficient opportunity to observe their immediate nurse manager’s leadership behavior and the hospital’s responses to patient-safety concerns.

Nurses were not eligible for recruitment if they were temporarily on leave during the data-collection period or worked in outpatient clinics, operating rooms, pediatric units, or administrative or support departments that did not provide direct adult inpatient care. Nurses holding exclusively managerial positions and not providing direct patient care were also excluded. These criteria were used to ensure that participants had sufficient exposure to inpatient clinical practice, their immediate nurse manager, patient-safety situations, and opportunities to speak up about clinical concerns.

### 2.4 Sample size

The initial sample size was estimated using Kish’s formula for cross-sectional survey studies:$n_{0}=\frac{Z^{2}p\left(1-p\right)}{d^{2}}$ where (n_0) represents the initial sample-size estimate, (Z) is the standard normal value corresponding to a 95% confidence level, (p) is the expected population proportion, and (d) is the accepted margin of error. Because no directly comparable previous study provided a reliable estimate of the proportion of nurses demonstrating speaking-up behavior in the study context, a conservative expected proportion of (p=.50) was used to maximize the required sample size. Using (Z=1.96), (p=.50), and (d=.05), the initial sample-size estimate was 384.16, which was rounded up to 385 nurses.

Because the total number of eligible nurses across the 13 participating hospitals was finite (N=1,100), the initial estimate was adjusted using the finite-population correction: $n=\frac{n_{0}}{1+\frac{n_{0}-1}{N}}$

After applying the finite-population correction, the minimum required sample size was 284.92, which was rounded up to 285 nurses. To compensate for an anticipated non-response or unusable-questionnaire rate of 10%, the minimum sample was inflated using the following formula:$\;n_{\text{target}}=\frac{285}{1-0.10}=316.67$*.*

The resulting target sample was rounded up to 317 nurses. However, 315 questionnaires were ultimately distributed during the available data-collection period. Of these, 303 were returned, yielding a response rate of 96.2%**.** Fifteen returned questionnaires were excluded because they were incomplete or contained response patterns that prevented valid scoring of one or more study instruments. Consequently, 288 questionnaires were included in the final analysis, yielding a usable response rate of 91.4% of the distributed questionnaires and 95.0% of the returned questionnaires. The final analytical sample exceeded the finite-population-adjusted minimum requirement of 285 nurses.

The sample-size calculation was intended to determine the recruitment target for the cross-sectional survey and was not a model-specific power calculation for the serial indirect-pathway analysis. Because no sufficiently comparable previous study provided reliable estimates for all component paths in the proposed serial model, a defensible a priori power calculation for the specific indirect effects was not available. Therefore, the adequacy of the final sample for detecting small individual or serial indirect associations cannot be assured. Indirect effects were reported with 95% bootstrap confidence intervals to describe their magnitude and precision, but bootstrapping was not considered a substitute for model-specific power analysis.

### 2.5 Sampling technique

A two-stage sampling strategy was used. First, purposive sampling was used to select hospitals that provided multiple adult inpatient services, such as medical, surgical, intensive care, obstetric, gynecological, orthopedic, burns, and open-heart care units. Pediatric hospitals, hospitals providing short-term care only, and hospitals that refused to approve data collection were excluded. The participating hospitals were therefore selected according to service availability, study eligibility criteria, and administrative approval rather than through probability sampling.

Second, the sample was allocated proportionally across the 13 participating hospitals according to the number of eligible nurses employed in each hospital. The total eligible nursing population across the participating hospitals was 1,100 nurses. The number of questionnaires allocated to each hospital was calculated using the following formula: nh = (Nh / 1100) * 315 where (nh) represents the number of questionnaires allocated to hospital (h), (Nh) represents the number of eligible nurses employed in that hospital, (N=1,100) represents the total eligible nursing population across the 13 hospitals, and (n=315) represents the total number of questionnaires distributed.

Within each hospital, eligible nurses who were available during the scheduled data-collection visits were approached consecutively until the proportionally allocated sample for that hospital was reached. Because participants were recruited according to their availability rather than selected randomly from complete staff lists, the nurse-level sampling procedure was classified as proportionate consecutive sampling rather than stratified random sampling.

Nurses who participated in the pilot study were excluded from the main study. Data-collection visits were conducted across different shifts and days of the week to improve the representation of nurses working under different schedules.

### 2.6 Data collection questionnaires

Data were collected using a structured, self-administered questionnaire consisting of five sections: demographic and work-related characteristics, perceived courageous leadership, perceived just culture, perceived psychological safety, and nurses’ speaking-up behavior.

#### 2.6.1 Demographic and work-related characteristics

The first section was developed by researchers to collect participants’ demographic and professional data, including hospital code, age, gender, educational qualification, marital status, years of nursing experience, current working unit, hospital type, previous patient safety training, and previous experience with incident reporting. Hospital codes rather than hospital names were used in the analytical dataset to protect institutional and participant confidentiality. No names, personnel numbers, telephone numbers, or other direct personal identifiers were collected.

#### 2.6.2 Courageous leadership questionnaire

Courageous leadership was assessed using an eight-item questionnaire developed by the researchers based on the courageous leadership framework proposed by Bangari and Prasad (2012). The questionnaire included four items addressing moral courage and four items addressing physical courage.

Participants were instructed to rate the courageous leadership behaviors of their immediate nurse manager. They were asked to indicate the extent to which each statement described the behavior of their immediate nurse manager. Participants rated each item on a five-point Likert scale ranging from 1 = strongly disagree to 5 = strongly agree. The total score ranged from 8 to 40, with higher scores indicating stronger nurses’ perceptions of their immediate nurse manager’s courageous leadership.

Given the nursing leadership context, the relevance and cultural appropriateness of both dimensions were reviewed by a panel of experts in nursing administration, patient safety, and research methodology. The experts evaluated the items for relevance, clarity, and contextual appropriateness rather than confirming the psychometric validity of the instrument. Minor wording modifications were made to improve contextual clarity. The modified wording retained the intended conceptual meaning of the original items.

The physical-courage items were interpreted as addressing leaders’ willingness to confront difficult workplace situations, intervene in unsafe practices, protect staff and patients, and take responsible action during challenging clinical or organizational circumstances. In the present study, the scale demonstrated acceptable internal consistency, with a Cronbach’s alpha coefficient of 0.87 for the overall scale.

#### 2.6.3 Just culture assessment tool

Perceived just culture was measured using the Just Culture Assessment Tool (JCAT) developed by Petschonek et al. (2013). The JCAT includes 27 items distributed across six dimensions: balance between a blame-free approach and accountability, feedback and communication, openness of communication, quality of the event-reporting process, continuous improvement, and trust. Items are rated on a seven-point Likert scale ranging from 1 = strongly disagree to 7 = strongly agree. The total score ranges from 27 to 189, with higher scores indicating stronger individual nurses’ perceptions of just culture within the hospital.

Items requiring reverse scoring were recoded according to the original scoring instructions before the total score was calculated. Although just culture is theoretically an organizational characteristic, the present study assessed nurses’ individual perceptions of that culture and did not aggregate scores to the hospital level.

In the present study, the 27-item scale demonstrated acceptable internal consistency, with a Cronbach’s alpha coefficient of 0.91 for the overall score.

#### 2.6.4 Team psychological safety scale

Psychological safety was assessed using Edmondson’s seven-item Team Psychological Safety Scale (Edmondson, 1999). The scale measures the extent to which nurses perceive their immediate work team as safe for interpersonal risk-taking, including expressing concerns, asking questions, acknowledging mistakes, requesting assistance, and challenging existing practices without fear of embarrassment, rejection, blame, or punishment.

Items were rated on a five-point Likert scale ranging from 1 = strongly disagree to 5 = strongly agree. Negatively worded items were reverse coded before calculating the overall score. The seven-item scores were summed, with higher scores indicating greater perceived psychological safety.

In the present study, the seven-item scale demonstrated acceptable internal consistency, with Cronbach’s alpha coefficient of 0.87 for the overall score. Because the scale was analyzed using individual nurses’ responses rather than aggregated team scores, the findings were interpreted as nurses’ individual perceptions of team psychological safety.

#### 2.6.5 Speaking up about patient safety questionnaire

Nurses’ speaking-up behavior was measured using the Speaking Up About Patient Safety Questionnaire (SUPS-Q), developed by Richard, Pfeiffer, and Schwappach (2021). The behavioral section of the SUPS-Q consists of 11 items distributed across three subdomains: perceived safety concerns, withholding voice, and speaking up.

Responses were measured using a five-point frequency scale referring to behavior during the previous four weeks: 1 = never (0 times), 2 = rarely (1–2 times), 3 = sometimes (3–5 times), 4 = often (6–10 times), and 5 = very often (more than 10 times). The perceived-safety-concerns subdomain contains three items, the withholding-voice subdomain contains four items, and the speaking-up subdomain contains four items.

The primary outcome in the present study was the four-item speaking-up subscale. Its score was calculated as the mean of the four constituent items, producing a possible score ranging from 1 to 5. Higher scores indicated more frequent speaking-up behavior during the previous four weeks. The perceived-safety-concerns and withholding-voice subdomains were not combined with the speaking-up items to create an overall behavioral score because the three subdomains represent distinct constructs.

During the original development of the SUPS-Q, Cronbach’s alpha coefficients were reported as 0.73 for perceived safety concerns, 0.76 for withholding voice, and 0.85 for speaking up. In this study, the corresponding Cronbach’s alpha coefficients were 0.80, 0.86, and 0.87, respectively.

### 2.7 Translation and cultural adaptation

The English versions of the study instruments were translated into Arabic using a forward–backward translation procedure. First, the instruments were independently translated from English into Arabic by bilingual experts with experience in nursing and healthcare research. The translated versions were then reviewed and reconciled to produce a preliminary Arabic version.

The preliminary Arabic versions were independently back-translated into English by a bilingual translator who had not participated in the forward translation and was blinded to the original English versions. The original and back-translated versions were compared to assess semantic, conceptual, idiomatic, and cultural equivalence.

The Arabic questionnaire was reviewed by a panel of experts in nursing administration, patient safety, and research methodology. The panel assessed the items for clarity, relevance, simplicity, and cultural appropriateness. The expert panel also examined whether the translated items retained the intended meaning of the original constructs. Minor linguistic and contextual modifications were made based on the panel’s recommendations without changing the number of items, response options, or scoring procedures.

The final Arabic versions were subsequently evaluated during the pilot study to assess clarity, comprehensibility, feasibility, and questionnaire completion time among clinical nurses.

### 2.8 Pilot study

A pilot study was conducted with 30 clinical nurses, representing approximately 10% of the initially estimated sample size, to assess the clarity, comprehensibility, feasibility, questionnaire format, and time required for completion. The pilot participants met the main study eligibility criteria and were recruited from Port Said General Hospital.

The pilot study also helped identify ambiguous wording, culturally unclear expressions, and formatting difficulties. Based on participants’ feedback, minor linguistic and formatting modifications were made to improve clarity without changing the number of items, response options, conceptual content, or scoring procedures of the instruments. The average time required to complete the questionnaire was approximately 12–15 minutes. Nurses who participated in the pilot study were excluded from the final study sample and were not included in the statistical analysis.

### 2.9 Data collection procedure

Before data collection, official administrative permission was obtained from the responsible authorities at the participating hospitals. The researchers met with nursing directors or authorized hospital representatives to explain the study purpose, eligibility criteria, sampling procedures, data-collection process, and ethical safeguards.

Data were collected from February 3 to February 26, 2026. Within each hospital, eligible nurses who were available during the scheduled data-collection visits were approached consecutively until the proportionally allocated sample for that hospital was reached. Data-collection visits were conducted at times that did not interfere with patient care.

The researchers explained the purpose of the study, the voluntary nature of participation, the confidentiality of responses, and participants’ right to decline or withdraw without penalty. Written informed consent was obtained from each nurse before questionnaire distribution.

Participants completed the self-administered questionnaire independently in a location that provided adequate privacy. Nurse managers and hospital administrators were not present while participants completed the questionnaires and did not have access to individual responses. The questionnaires were collected by the researchers on the same day whenever possible. When participants required additional time, the completed questionnaires were collected later according to the circumstances of the clinical unit.

Completed questionnaires were reviewed for completeness without attempting to identify individual participants. Fifteen returned questionnaires were excluded because they were incomplete or contained response patterns that prevented valid scoring of one or more study instruments. The remaining 288 questionnaires were coded and included in the final statistical analysis.

### 2.10 Ethical considerations

Ethical approval was obtained from the Standing Committee of Bioethics Research at Prince Sattam bin Abdulaziz University (approval no. SCBR-112-2026) on January 21, 2026. Administrative approval was also obtained from the responsible authorities at each participating hospital.

The study was conducted in accordance with the ethical principles of the Declaration of Helsinki. Participants received written and verbal information explaining the study purpose, procedures, voluntary nature of participation, and confidentiality safeguards. Written informed consent was obtained from all participants before questionnaire completion.

Participants were informed that they could decline participation or withdraw before submitting the questionnaire without penalty or any effect on their employment. Nurse managers and hospital administrators were not informed of individual participation decisions and did not have access to completed questionnaires or individual responses.

Anonymity was maintained by assigning numerical codes to the questionnaires and avoiding the collection of names, personnel numbers, telephone numbers, or other direct personal identifiers. Hospital and clinical-unit information was treated as potentially identifying contextual data and was reported only in aggregate form.

Completed questionnaires were stored securely, and electronic data were maintained in password-protected files accessible only to the research team. All collected information was used solely for research purposes, and the findings were reported in aggregate form to prevent the identification of individual participants.

### 2.11 Data analysis

Data were analyzed using IBM SPSS Statistics, version 27. Descriptive statistics, including frequencies, percentages, means, and standard deviations, were used to summarize participant characteristics and study variables. Cronbach’s alpha was used to assess the internal consistency of the study instruments.

An independent-samples t-test was used to examine differences according to gender, whereas one-way analysis of variance was used to examine differences according to age group, marital status, educational level, and years of nursing experience. Pearson’s correlation coefficients were used to examine associations among perceived courageous leadership, just culture, psychological safety, and speaking-up behavior.

Before regression analysis, linearity, residual distributions, homoscedasticity, influential observations, and multicollinearity were examined. The prespecified serial indirect-pathway model was tested using PROCESS Macro version 5, Model 6. Perceived courageous leadership was entered as the independent variable (X), perceived just culture as the first intervening variable (M1), psychological safety as the second intervening variable (M2), and the mean score of the four speaking-up items as the dependent variable (Y).

HC3 heteroskedasticity-consistent standard errors were used for the regression paths. Indirect effects were estimated using 5,000 percentile bootstrap resamples and 95% bootstrap confidence intervals. An indirect effect was considered statistically significant when its 95% bootstrap confidence interval did not include zero. Statistical significance for the remaining analyses was set at p < .05, two-tailed.

Because the exposure, intervening variables, and outcome were measured concurrently, the PROCESS findings were described as statistical indirect associations or indirect pathways rather than evidence of causal mediation.

## 3 Results

A total of 288 nurses were included in the final analysis. Of these, 175 nurses (60.8%) were aged 30 years or younger, 103 (35.8%) were aged 31–40 years, 8 (2.8%) were aged 41–50 years, and 2 (0.7%) were aged 51 years or older. Most participants were female (n = 208, 72.2%), whereas 80 participants (27.8%) were male.

Regarding marital status, 79 nurses (27.4%) were single, 172 (59.7%) were married, 22 (7.6%) were divorced, and 15 (5.2%) were widowed. With respect to educational level, 122 nurses (42.4%) had technical nursing education, 110 (38.2%) held a bachelor’s degree, 46 (16.0%) held a master’s degree, and 10 (3.5%) held a PhD.

In terms of nursing experience, 92 nurses (31.9%) had 1–5 years of experience, 173 (60.1%) had 6–10 years, 20 (6.9%) had 11–15 years, and 3 (1.0%) had 16 years or more of experience.

Table 1 presents differences in courageous leadership, just culture, psychological safety, and nurses’ speaking-up behavior according to participants’ demographic and professional characteristics. An independent-samples t-test was used to examine differences by gender, whereas one-way analysis of variance was used to examine differences according to age group, marital status, educational level, and years of nursing experience.

**Table 1:** Differences in study variables by participants’ demographic and professional characteristics (N = 288).

Demographic characteristics	Group	N	Courageous leadership			Just culture			Psychological safety			Speaking-up behavior		
			Mean	SD	Test of sig.	Mean	SD	Test of sig.	Mean	SD	Test of sig.	Mean	SD	Test of sig.
**Age**	≤30 years	175	26.07	4.06	F = 0.145 *p* = .933	100.19	9.70	F = 0.225 *p* = .879	24.33	2.86	F = 0.951 *p* = .416	3.46	0.66	F = 0.851 *p* = .467
	31–40 years	103	26.28	4.22		100.37	9.67		24.20	2.72		3.54	0.66	
	41–50 years	8	26.88	2.70		98.88	7.12		24.63	2.72		3.75	0.40	
	≥51 years	2	26.50	2.12		105.00	4.24		27.50	2.12		3.75	0.00	
**Gender**	Male	80	26.09	3.66	*t* = −0.213 *p* = .831	99.90	8.83	*t* = −0.384 *p* = .701	24.38	3.02	t = 0.221 *p* = .825	3.52	0.68	*t* = 0.435
*p* = .664														
	Female	208	26.20	4.23		100.38	9.87		24.29	2.72		3.48	0.65	
**Marital status**	Single	79	26.52	3.78	F = 0.942 *p* = .421	100.18	9.18	F = 2.078 *p* = .103	24.14	2.43	F = 2.349 *p* = .073	3.42	0.57	F = 0.776 *p* = .508
	Married	172	26.14	4.22		100.65	9.45		24.48	2.96		3.54	0.67	
	Divorced	22	26.23	3.75		95.73	11.08		23.05	2.61		3.40	0.70	
	Widowed	15	24.60	4.26		102.67	9.73		25.20	2.57		3.50	0.83	
**Educational level**	Technical	122	26.44	4.13	F = 0.562 *p* = .641	99.25	9.78	F = 0.995 *p* = .395	24.14	2.80	F = 0.903 *p* = .440	3.51	0.65	F = 0.236
*p* = .871														
	Bachelor	110	25.84	4.08		100.57	9.26		24.50	2.84		3.49	0.64	
	Master	46	26.39	4.16		101.67	9.96		24.11	2.61		3.43	0.74	
	PhD	10	25.50	2.84		102.40	8.60		25.40	3.20		3.60	0.49	
**Experience years**	1–5 years	92	26.10	4.12	F = 1.786 *p* = .150	102.14	9.67	F = 2.061 *p* = .106	24.75	2.69	F = 1.958 *p* = .121	3.48	0.65	F = 0.853 *p* = .466
	6–10 years	173	26.23	4.09		99.43	9.27		24.13	2.74		3.48	0.67	
	11–15 years	20	25.25	3.55		98.15	11.07		23.60	3.68		3.61	0.58	
	≥16 years	3	31.00	0.00		103.33	9.02		26.33	1.15		4.00	0.43	

**Note**. Values are presented as mean and standard deviation (SD). Independent samples t-test was used for gender; one-way ANOVA was used for age, marital status, educational level, and experience years. No statistically significant differences were found across demographic groups for all study variables (p > .05).

No statistically significant differences were identified in courageous leadership, just culture, psychological safety, or speaking-up behavior across age groups, gender, marital status, educational levels, or years of nursing experience (all p > .05). These findings indicate that participants’ perceptions of the main study variables were generally comparable across the examined demographic and professional groups. Nevertheless, subgroup comparisons should be interpreted cautiously because some categories included very small numbers of participants, particularly nurses aged 51 years or older, nurses holding a PhD, and nurses with 16 years or more of experience.

Table 2 presents the descriptive statistics for the main study variables. The mean courageous leadership score was 26.17 (SD = 4.07), with observed scores ranging from 16 to 38 within a possible range of 8–40. The mean just culture score was 100.25 (SD = 9.58), with observed scores ranging from 78 to 125 within a possible range of 27–189. Psychological safety had a mean score of 24.32 (SD = 2.80), with observed scores ranging from 19 to 34 within a possible range of 7–35. The mean speaking-up behavior score was 3.49 (SD = 0.65), with observed scores ranging from 1.50 to 5.00 within a possible range of 1–5.

**Table 2:** Descriptive Statistics of the Main Study Variables (N = 288).

Variable	No. of items	Possible range	Actual range	Mean	SD
**Courageous leadership**	8	8–40	16–38	26.17	4.07
**Just culture**	27	27–189	78–125	100.25	9.58
**Psychological safety**	7	7–35	19–34	24.32	2.80
**Speaking-up behavior**	4	1-5	1.5 –5	3.49	0.65

**Note.** SD = standard deviation.

Table 3 presents Pearson’s correlation coefficients among perceived courageous leadership, just culture, psychological safety, and speaking-up behavior. Courageous leadership was positively associated with just culture, psychological safety, and speaking-up behavior. Just culture was strongly and positively associated with psychological safety and was weakly but significantly associated with speaking-up behavior. Psychological safety was not significantly associated with speaking-up behavior.

**Table 3:** Correlation Matrix among the Main Study Variables (N = 288).

Variable	Courageous leadership	Just culture	Psychological safety
**Courageous leadership**	1		
**Just culture**	.226⁎⁎	1	
**Psychological safety**	.288⁎⁎	.765⁎⁎	1
**Speaking-up behavior**	.214⁎⁎	.121*	.071

**Note**.

⁎ p < .05

⁎⁎ p < .01, two-tailed.

Table 4 presents the serial indirect pathway analysis examining whether just culture and psychological safety statistically accounted for part of the association between courageous leadership and nurses’ speaking-up behavior. Courageous leadership was positively associated with just culture. In the model predicting psychological safety, both courageous leadership and just culture were significant positive predictors.

**Table 4:** Serial Indirect Pathway Analysis Using PROCESS Model 6 (N = 288).

**Part A. Regression paths**						
**Outcome variable**	**Predictor**	**B**	**SE(HC3)**	**t**	**p**	**95% CI**
**Just culture**	Courageous leadership	0.5312	0.1284	4.1367	< .001	(0.2785, 0.7840)
**Psychological safety**	Courageous leadership	0.0833	0.0270	3.0803	.0023	(0.0301, 0.1366)
	Just culture	0.2155	0.0124	17.4024	< .001	(0.1912, 0.2399)
**Speaking-up behavior**	Courageous leadership	0.0338	0.0096	3.5061	< .001	(0.0148, 0.0528)
	Just culture	0.0107	0.0060	1.7983	.0732	(-0.0010, 0.0225)
	Psychological safety	-0.0257	0.0204	-1.2626	.2078	(-0.0658, 0.0144)
**Part B. Bootstrap indirect effects**						
**Indirect pathway**	**Effect**	**Boot SE**	**Boot LLCI**	**Boot ULCI**	**Result**	
**Total indirect effect**	0.0006	0.0029	-0.0051	0.0065	Not significant	
**CL → JC → Speaking-up behavior**	0.0057	0.0035	-0.0004	0.0134	Not significant	
**CL → Psychological safety → Speaking-up behavior**	-0.0021	0.0019	-0.0065	0.0009	Not significant	
**CL → JC → Psychological safety → Speaking-up behavior**	-0.0029	0.0024	-0.0083	0.0015	Not significant	
**Part C. Total and direct effects**						
**Effect**	**B**	**SE(HC3)**	**t**	**p**	**95% CI**	
**Total effect of CL on speaking-up behavior**	0.0344	0.0095	3.6409	< .001	(0.0158, 0.0531)	
**Direct effect of CL on speaking-up behavior**	0.0338	0.0096	3.5061	< .001	(0.0148, 0.0528)	

**Note.** CL = courageous leadership; JC = just culture; SE = standard error; CI = confidence interval; LLCI = lower limit of the confidence interval; ULCI = upper limit of the confidence interval. HC3 heteroskedasticity-consistent standard errors were used for the regression paths. Indirect effects were estimated using 5,000 percentile bootstrap samples. An indirect effect was considered statistically significant when its 95% bootstrap confidence interval did not include zero.

In the final model predicting speaking-up behavior, courageous leadership remained a significant positive predictor. However, neither just culture nor psychological safety was significantly associated with speaking-up behavior after accounting for the other variables.

The total effect of courageous leadership on speaking-up behavior was significant, and its direct effect remained significant after the proposed intervening variables were entered. However, the total indirect effect was not significant because its 95% bootstrap confidence interval included zero.

The specific indirect effects through just culture (effect = 0.0057, BootSE = 0.0035, 95% bootstrap CI [−0.0004, 0.0134]), through psychological safety (effect = −0.0021, BootSE = 0.0019, 95% bootstrap CI [−0.0065, 0.0009]), and through the serial pathway involving just culture followed by psychological safety (effect = −0.0029, BootSE = 0.0024, 95% bootstrap CI [−0.0083, 0.0015]) were also nonsignificant. Thus, the findings supported a direct positive association between courageous leadership and speaking-up behavior but did not support the proposed individual or serial indirect pathways through just culture and psychological safety.

Regarding the study hypotheses, H1a, H1b, H1c, and H2 were supported. H3a received bivariate support only: just culture was positively correlated with speaking-up behavior, but its adjusted association in the final regression model was not statistically significant. H3b, H4, H5, and H6 were not supported.

## 4 Discussion

This study examined the association between courageous leadership and nurses’ speaking-up behavior and explored the indirect roles of just culture and psychological safety among clinical nurses in thirteen hospitals in Port Said, Egypt. The findings provided partial support for the proposed model. Courageous leadership was positively associated with speaking-up behavior, just culture, and psychological safety. Just culture was also positively associated with psychological safety. However, neither psychological safety nor just culture showed a significant indirect role in the association between courageous leadership and speaking-up behavior. In addition, the proposed serial indirect pathway through just culture and psychological safety was not statistically significant. Given the cross-sectional design, these findings should be interpreted as associations and statistical indirect pathways rather than causal mechanisms.

Courageous leadership was positively associated with nurses’ speaking-up behavior, supporting the hypothesis that leadership behaviors characterized by ethical action, openness, and willingness to confront unsafe practices are related to greater patient safety communication. This finding is consistent with previous literature indicating that courageous, ethical, and values-based leadership can support open communication and reduce silence in clinical settings (Althobaiti, 2026; Bangari & Prasad, 2012; Huang et al., 2025; Meng & Guo, 2025). Nurses who perceived their leaders as willing to address unsafe practices and act according to ethical principles reported higher speaking-up behavior. Courageous leadership also remained directly associated with speaking-up behavior after including just culture and psychological safety, suggesting that other factors, such as leader accessibility, role modeling, trust, professional responsibility, psychological empowerment, and moral courage, may also contribute to nurses’ willingness to raise safety concerns (Lee & Cho, 2026; Mohammadi et al., 2022; Wang et al., 2024; Yılmaz & Özbek Güven, 2024). The persistence of this direct association indicates that courageous leadership may influence nurses’ speaking-up behavior through mechanisms not fully captured by the proposed mediators.

Courageous leadership was also positively associated with just culture. This finding is theoretically consistent with the role of nurse leaders in shaping staff perceptions of fairness, accountability, transparency, and responses to error. Courageous leaders may be more willing to challenge blame-oriented practices, respond constructively to mistakes, and encourage learning from adverse events rather than punishment. These behaviors are central to just culture, which emphasizes balanced accountability and system improvement (Marx, 2001; Petschonek et al., 2013; Robichaux & Vittone, 2023). This finding is also consistent with studies linking supportive safety cultures and leadership practices with reduced silence and improved error reporting among nurses (Badran et al., 2026; Ibrahim El-Sayed et al., 2024). However, because the study was cross-sectional, this association should not be interpreted as evidence that courageous leadership directly produced stronger just culture perceptions.

Just culture was strongly and positively associated with psychological safety. This suggests that nurses who perceived higher fairness, trust, open communication, and learning-oriented responses to error also felt safer to express concerns within their teams. This finding is theoretically plausible because organizational-level fairness may strengthen team-level confidence that speaking up will not result in blame, embarrassment, or punishment. Although just culture and psychological safety were closely related, they were retained as distinct constructs because just culture reflects broader organizational fairness and accountability, whereas psychological safety reflects the immediate interpersonal climate within teams (Edmondson, 1999; Ito et al., 2022; O’Donovan et al., 2021). Nevertheless, the strong relationship between the two variables suggests possible shared variance and should be considered when interpreting the regression and indirect-pathway findings.

In contrast to the original hypothesis, psychological safety was not significantly associated with nurses’ speaking-up behavior at the bivariate level. It also did not significantly predict speaking-up behavior after courageous leadership and just culture were included in the final regression model. This finding differs from Edmondson’s theoretical proposition that team members are more likely to ask questions, admit mistakes, challenge unsafe decisions, and raise concerns when they feel psychologically safe (Edmondson, 1999). It also differs from previous healthcare research linking psychological safety with voice behavior, collaborative problem-solving, and safety improvement (Bahadurzada et al., 2024; O’Donovan et al., 2021; Weiss et al., 2023).

Several explanations may account for this unexpected result. First, psychological safety may be necessary but not sufficient for speaking-up behavior, particularly in strongly hierarchical healthcare environments where nurses may still anticipate professional, relational, or organizational consequences. Second, the strong association between just culture and psychological safety may have reduced the unique contribution of psychological safety in the multivariable model. Third, nurses may distinguish between feeling generally safe within their teams and being willing to raise concerns in situations involving senior physicians, managers, or potential patient harm. Therefore, the absence of a significant association should not be interpreted as evidence that psychological safety is unimportant, but rather that it did not independently explain variation in speaking-up behavior in the present sample.

The indirect pathway findings provide further insight into the proposed model. Just culture alone did not show a significant indirect association between courageous leadership and speaking-up behavior, although it was positively correlated with speaking-up behavior at the bivariate level. Furthermore, just culture did not significantly predict speaking-up behavior after courageous leadership and psychological safety were entered into the final model. This pattern suggests that perceptions of organizational fairness and learning-oriented responses to error were related to speaking up at the bivariate level, but their unique contribution became nonsignificant after accounting for the other variables. This may indicate that just culture is not directly translated into speaking-up behavior or that its influence operates through additional organizational or interpersonal mechanisms not examined in this study.

Psychological safety did not show a significant indirect role in the association between courageous leadership and speaking-up behavior. Although courageous leadership significantly predicted psychological safety, psychological safety did not significantly predict speaking-up behavior. Consequently, the bootstrap confidence interval for this specific indirect effect included zero. This finding suggests that the positive association between courageous leadership and speaking-up behavior was not statistically explained by psychological safety in the present dataset.

The proposed serial indirect pathway through just culture and psychological safety was also not statistically significant. Courageous leadership was positively associated with just culture, and just culture was strongly associated with psychological safety; however, psychological safety was not significantly associated with speaking-up behavior. As a result, the full sequence from courageous leadership to just culture, then to psychological safety, and finally to speaking-up behavior was not supported. The significant relationships among the earlier parts of the pathway indicate that leadership, organizational culture, and team climate remain interconnected, but these relationships did not jointly account for nurses’ speaking-up behavior.

The nonsignificant total indirect effect, together with the significant direct effect of courageous leadership, indicates that the observed association between courageous leadership and speaking-up behavior was primarily direct. This finding may reflect the immediate influence of leader behavior on nurses’ decisions to raise concerns. For example, leaders who visibly challenge unsafe practices, invite dissenting views, respond respectfully to concerns, and protect staff from retaliation may affect speaking-up decisions without necessarily changing broader perceptions of just culture or psychological safety.

No statistically significant differences were found in courageous leadership, just culture, psychological safety, or speaking-up behavior according to age, gender, marital status, educational level, or years of nursing experience. This suggests that perceptions of the main study variables were relatively similar across demographic and professional groups in the present sample. These findings may indicate that speaking-up behavior and safety-related perceptions are shaped more by leadership, organizational culture, and team climate than by individual demographic characteristics (Lee & Kwon, 2024; Yilmaz, 2025). However, this interpretation should be made cautiously because some demographic subgroups were small, particularly nurses aged 51 years or older, nurses with PhD qualifications, and nurses with 16 years or more of experience.

Overall, the findings highlight courageous leadership as the factor most consistently associated with nurses’ speaking-up behavior. Just culture and psychological safety were strongly interrelated and were both associated with courageous leadership, but neither demonstrated a significant indirect role in the relationship between courageous leadership and speaking-up behavior. For nursing administration, this means that promoting speaking up requires visible and direct leadership behaviors, including inviting concerns, responding constructively, protecting staff from retaliation, and demonstrating willingness to confront unsafe practices. At the same time, fair organizational responses to error and psychologically safe team environments remain important contextual conditions, even though they did not statistically account for the leadership–speaking-up association in this study.

### 4.1 Limitations of the study

Several limitations should be considered. First, the cross-sectional design limits causal inference and prevents confirmation of the temporal ordering assumed in the serial pathway model. Second, the use of self-reported questionnaires may introduce common method bias and social desirability bias. Third, the study was conducted in Port Said, Egypt, which may limit generalizability to other settings. Fourth, the strong association between just culture and psychological safety raises questions about possible conceptual overlap and shared variance, which may have reduced their unique predictive contributions in the final model. Fifth, the nonsignificant indirect effects suggest that other mechanisms, such as leader accessibility, trust in management, fear of retaliation, moral courage, professional hierarchy, or prior experiences of speaking up, may better explain the association between courageous leadership and speaking-up behavior.

Finally, nurses were nested within hospitals and clinical units, but the analysis did not explicitly model this organizational clustering. Although HC3 heteroskedasticity-consistent standard errors were used, these do not fully address possible within-hospital dependence. Consequently, the precision of the regression estimates may have been affected. Future studies should use multilevel models, cluster-robust standard errors, or multilevel structural equation modeling when sufficient numbers of hospitals and units are available.

### 4.2 Implications for nursing administration and patient safety practice

Leadership development programs should include courageous leadership, moral courage, ethical decision-making, and constructive responses to error (Huang et al., 2025; Meng & Guo, 2025). Emphasis should be placed on observable leader behaviors that directly encourage speaking up, such as actively soliciting concerns, acknowledging uncertainty, responding without defensiveness, providing feedback after concerns are raised, and protecting nurses from interpersonal or organizational retaliation.

Hospitals should also strengthen just culture by promoting fair accountability, transparent feedback, and learning-oriented responses to incidents (Badran et al., 2026; Petschonek et al., 2013; Robichaux & Vittone, 2023). At the team level, structured debriefings, communication workshops, speaking-up simulation, and psychological safety training may help nurses feel more able to express patient safety concerns (Hur et al., 2025; Silva et al., 2025). However, the present findings indicate that improving just culture or psychological safety alone may not automatically result in greater speaking-up behavior. These initiatives should therefore be combined with explicit reporting pathways, anti-retaliation policies, leader accountability, and timely organizational responses to staff concerns.

Because demographic differences were not significant, interventions should focus mainly on organizational and team-level conditions rather than specific demographic subgroups. Future interventions should also be evaluated using longitudinal or experimental designs to determine whether changes in leadership behavior, just culture, and psychological safety are followed by measurable improvements in speaking-up behavior.

## 5 Conclusion

This study found that courageous leadership was positively associated with nurses’ speaking-up behavior, just culture and psychological safety among clinical nurses in hospitals in Port Said, Egypt. While just culture was strongly associated with psychological safety. However, neither just culture nor psychological safety showed a significant indirect role in the association between courageous leadership and speaking-up behavior. The proposed serial indirect pathway through just culture and psychological safety was also not supported.

The findings therefore suggest that courageous leadership was related to speaking-up behavior primarily through a direct association rather than through the proposed mediating variables. Just culture and psychological safety remain important and closely related features of the organizational and team environment, but they did not statistically explain why nurses who perceived greater courageous leadership reported higher speaking-up behavior. Nursing leaders should therefore combine efforts to build fair and psychologically safe environments with direct behaviors that invite, reinforce, and protect speaking up. Because the study was cross-sectional, the findings should be interpreted as associations rather than causal effects.

## CRediT authorship contribution statement

**Ali D. Abousoliman:** Writing – review & editing, Writing – original draft, Visualization, Validation, Supervision, Software, Funding acquisition, Formal analysis, Data curation, Conceptualization. **Heba Gamal Elgamal:** Resources, Project administration, Methodology, Formal analysis, Conceptualization.

## Declaration of competing interest

The authors declare no competing interests.

## Data Availability

The data that support the findings of this study are available from the corresponding author upon reasonable request.
